# Deciphering the Structure–Immunomodulatory Function Relationships of Homopolysaccharides

**DOI:** 10.3390/nu18111782

**Published:** 2026-05-31

**Authors:** Gege Hu, Bingyu Yang, Han Song, Yuehan Zeng, Zhuoting Zhang, Yuwen Li, Jian Zhang, Fenghuan Wang

**Affiliations:** 1 School of Light Industry Science and Engineering, Beijing Technology and Business University (BTBU), Beijing 100048, China; hugege0819@163.com; 2 Beijing Advanced Innovation Center for Food Nutrition and Human Health, Beijing Technology and Business University (BTBU), Beijing 100048, China; 19835230803@163.com (B.Y.); songh0107@163.com (H.S.); zeng55272024@163.com (Y.Z.); zzt17303410361@163.com (Z.Z.); 18407718130@163.com (Y.L.); jian_zh@outlook.com (J.Z.); 3 Beijing Engineering and Technology Research Center of Food Additives, Beijing Technology and Business University (BTBU), Beijing 100048, China

**Keywords:** homopolysaccharides (HoPSs), structure–activity relationship, immunomodulatory activity, structural characterization, signal pathway

## Abstract

Natural homopolysaccharides (HoPSs), composed of a single monosaccharide type, are increasingly recognized as bioactive macronutrients with broad relevance to nutrition and health. This review summarizes the extraction, structural characterization, and structure-immunomodulatory activity relationships of HoPSs. Drawing on a comprehensive synthesis of existing studies, we integrate current knowledge into a unified hierarchical framework of HoPS structure–function relationships. This framework organizes the literature into three hierarchical levels, including primary structural recognition, mid-level regulatory mechanisms, and functional refinement, while integrating key determinants such as molecular weight, glycosidic linkages, chain conformation, branching, and chemical modifications. By bridging structural glycomics and nutritional immunology, this framework synthesizes current evidence and provides a structured reference for future investigations. HoPSs exert well-established anti-infection and anti-inflammatory effects, alongside important nutritional and metabolic benefits. These outcomes are supported by evidence from cellular receptor signaling (e.g., TLRs, Dectin-1; NF-κB, MAPK pathways), gut microbiota remodeling, and metabolite network interactions. Finally, we discuss current research gaps, particularly in fine structural analysis and multidimensional mechanistic studies, and propose future directions based on precise structural elucidation, multidimensional structure–activity relationship modeling, and interdisciplinary integration. This review aims to bridge structural glycomics with human nutritional immunology, providing a theoretical basis for the structural optimization, immune activity enhancement, and functional food development of natural HoPSs to promote their industrial application in medicine, nutrition, and health.

## 1. Introduction

Polysaccharides, or polyglycans, are high-molecular-weight carbohydrates composed of more than ten monosaccharide units linked by glycosidic bonds formed through dehydration condensation. These biopolymers exhibit a broad molecular weight distribution, typically ranging from tens of thousands to tens of millions Da [1]. Polysaccharides are widely distributed in nature and have been identified in herbal plant seeds, stem and leaf tissues, animal body fluids, and cell walls, as well as in bacteria, yeast, fungi, and other microorganisms [2]. In recent years, increasing studies have demonstrated that polysaccharides possess various pharmacological activities, such as antibacterial, antitumor, antiviral, and antioxidant effects [3]. The structure of polysaccharides serves as the foundation for their biological functions, yet related research lags behind other macromolecules [4]. Compared with these, polysaccharides have more monosaccharide types, connection modes and intramolecular hydrogen bonding formation modes and the possibility for functional group modification, which makes the study of the advanced structure of polysaccharides more difficult. This complexity endows polysaccharides with rich structural information, and the presence or absence of specific structures may have significant impacts on the differences between health and disease [5]. Therefore, a comprehensive and scientific understanding of polysaccharide structure is of great value for both fundamental research and applied development.

Polysaccharides are mainly categorized into two types: homopolysaccharides (HoPSs) and heteropolysaccharides (HePSs) [6]. While HePSs represent the majority of identified glycans (>70%) in microbial and algal sources, HoPSs constitute the bulk of global carbohydrate mass due to their role as universal structural (e.g., cellulose, chitin) and storage (e.g., starch, glycogen) polymers [7,8]. It is estimated that cellulose alone accounts for over 40% of the total organic carbon in the biosphere, making HoPSs the most abundant biopolymers on Earth. Despite this prevalence, the literature has historically prioritized the chemical characterization of these dominant polymers, whereas systematic reviews linking their specific structural dimensions to immunomodulatory mechanisms remain scarce. We specifically focus on HoPSs in this review because their monomeric homogeneity provides a distinct advantage for deciphering structure–activity relationships. Unlike the combinatorial complexity of HePSs, the defined nature of HoPSs allows for a more precise evaluation of how specific topological parameters (e.g., glycosidic linkage type, degree of branching, and chain conformation) directly dictate biological function. This focused approach is essential to address the current knowledge gap regarding the synergistic governance of immune outcomes by multiple structural dimensions.

HoPSs are high-molecular-weight polymers formed by condensation of identical monosaccharide units, yielding only one type of monosaccharide upon hydrolysis, such as starch, glycogen, or cellulose [6]. The composition of the backbone sugar units directly determines their classification. For example, the structure of amylose is connected by an α-(1→4)-glycosidic bond, while amylopectin forms branches through α-(1→6)-glycosidic bonds [9]. With the development of various analytical techniques, the advanced structures of HoPSs have been extensively studied, providing reliable evidence for their structural characterization. As a single analytical method is often insufficient to unveil the complete structure, data integration from multiple techniques is essential to construct a comprehensive architectural picture.

Naturally occurring HoPSs exhibit remarkable diversity in structural characteristics, including monosaccharide composition, glycosidic bond types, degree of polymerization, degree of branching, spatial conformation and solubility [10]. This structural diversity is the fundamental determinant of the potency, direction, and mechanism of their immunomodulatory activities. Studies have confirmed that natural HoPSs with suitable structures can modulate innate and adaptive immunity by directly activating receptors on immune cells (e.g., macrophages, dendritic cells, and lymphocytes), regulating immune signaling pathways, or indirectly modulating gut microbiota structure and mediating the gut microbiota and mucosal immune axis [11,12,13]. Deciphering the structure–activity relationship is therefore a prerequisite for elucidating immunomodulatory mechanisms and developing novel immunomodulatory agents [14]. However, current research is often limited to analyzing the immunoactivity of HoPSs from a single structural perspective or focusing on a specific polysaccharide in isolation. There is a lack of systematic investigation into how multiple structural dimensions synergistically govern immune outcomes. Furthermore, the association networks between structural features and immunoactivity remain inadequately mapped, and the precise immunomodulatory targets and signaling pathways for many HoPSs are yet to be fully elucidated. These knowledge gaps significantly hinder the precise development and application of natural HoPSs in immunomodulation.

Beyond their structural complexity, homopolysaccharides (HoPSs) are vital dietary components, functioning as fibers and energy sources prevalent in cereals, fungi, and vegetables [15,16]. Typical daily intakes range from 3 to 10 g, with higher doses recommended for specific health benefits [17]. Unlike drugs, the biological fate of these polysaccharides is determined by their structures, which determine their fermentation ability in the gut microbiota and subsequent immunomodulatory cascades in vivo [18]. Crucially, food processing markedly alters their molecular weight and branching, directly impacting bioaccessibility and colonic fermentation kinetics [19]. As these glycans resist upper GI hydrolysis, their physiological fate is dictated by interactions with the gut ecosystem, where microbial fermentation shapes the intestinal environment and underpins systemic immune resilience [20]. This structural diversity provides a robust foundation for functional food applications. Structurally defined HoPSs are increasingly incorporated into fortified foods and medical nutrition formulas [21]. Furthermore, emerging strategies focus on tailoring HoPS type, dose, and delivery to individual dietary patterns, representing a promising frontier in precision nutrition aimed at enhancing immune health.

**Figure 1:**
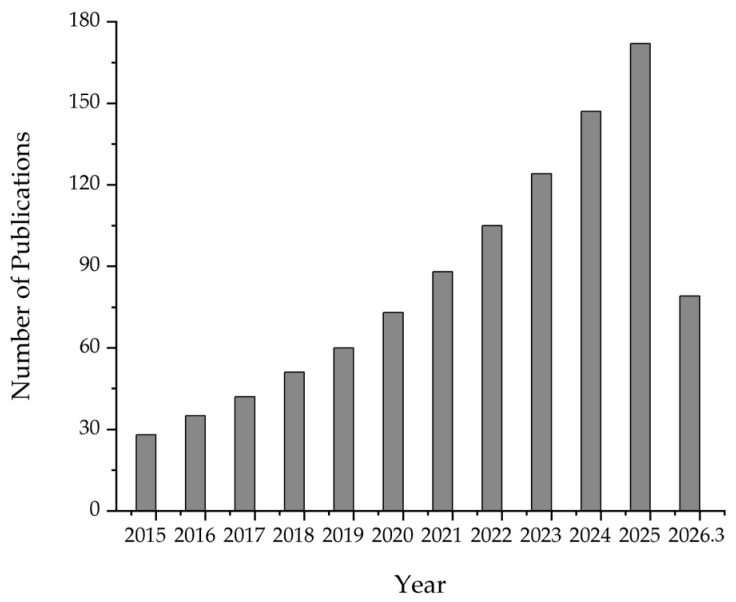
Annual publication trend in HoPS structure–function relationships.

A systematic literature survey was conducted across Web of Science, PubMed, and Scopus for publications up to March 2026. The search strategy used keyword combinations including HoPSs, glycan, conformation, and immunomodulation to identify studies on the structural features and biological functions of HoPSs. The relevance and quality of the retrieved articles were subsequently assessed based on their methodological soundness and direct contribution to the topic, leading to the exclusion of reviews, conference abstracts, and studies on chemically synthesized polymers. Figure 1 illustrates the annual number of publications related to homopolysaccharide (HoPS) structure–function relationships indexed in Web of Science (2015–2026.5). The data depict a rapid increase in research output, reflecting growing scientific interest in the structural basis of HoPS bioactivity and their nutritional applications.

Based on the above, this article systematically reviews the core structural features of natural HoPSs (e.g., monosaccharide types, glycosidic bond types, degree of polymerization, etc.) and summarizes the structure–activity relationships between various structural dimensions and immunomodulatory activities. We analyze how specific structural characteristics dictate immunomodulatory mechanisms and compare the structure-dependent immune activities across different types of HoPSs. The immune regulatory process of the HoPSs was also summarized, elucidating their internal process of immune regulation through regulating key links such as immune cell activation, cytokine secretion, and signal pathway transduction. It also bridges structural glycomics and nutritional immunology, evaluating how HoPS physicochemistry translates into gut barrier reinforcement and systemic immune homeostasis for functional food applications. Finally, in light of current research shortcomings, we propose future directions aimed at providing a theoretical foundation for the structural optimization of natural HoPSs, a deeper understanding of their mechanisms, and the development of novel polysaccharide-based immunomodulators. This endeavor is expected to promote the efficient utilization of HoPSs in food, pharmaceutical, and nutraceutical industries.

## 2. Extraction and Structural Analysis of HoPSs

### 2.1. Impact of Extraction Methods and Conditions on the Structure of HoPSs

Common methods for extracting HoPSs include hot-water, alkaline, and enzymatic extraction, as well as techniques assisted by microwaves or ultrasound. The extraction method significantly influences the structural characteristics of the obtained polysaccharides, including molecular weight distribution, branching degree, and substituent groups. Traditional hot-water extraction is a mild process that minimizes structural degradation, though it may yield products with relatively lower molecular weights [22]. Acid or alkaline extraction is typically employed for insoluble polysaccharides but requires stringent control of conditions (e.g., concentration, temperature, and duration) to prevent glycosidic bond cleavage and a consequent reduction in molecular weight [23]. Enzymatic extraction offers high specificity by utilizing enzymes that cleave targeted bonds, thereby providing superior structural preservation and adaptability to diverse polysaccharide types [24]. Ultrasound- and microwave-assisted methods enable efficient extraction under relatively mild conditions; however, careful optimization of parameters (e.g., power, time) is essential to minimize the risk of structural damage and to obtain products with higher molecular weight and preserved integrity [25].

Beyond the choice of extraction method, process parameters can profoundly alter HoPS structure. Elevated temperatures during extraction or processing, especially above 150 °C, can accelerate glycosidic bond hydrolysis and cause a marked reduction in molecular weight [23]. This breakdown disrupts intra- and intermolecular hydrogen-bond networks, which can cause the collapse of secondary structures and the loosening or even depolymerization of higher-order assemblies [26]. Similarly, highly acidic or alkaline conditions can selectively cleave specific glycosidic linkages, disrupt hydrogen bonding, and completely dismantle secondary and tertiary structures [27]. While prolonged extraction times generally exacerbate structural damage [19], the optimal duration is not universal. For instance, extraction yields of exopolysaccharides from Pediococcus acidilactici peak at 18 h [28], whereas those of Aureobasidium pullulans mannan are maximized within only 3 h [29]. Therefore, the extraction time must be tailored to the specific microorganism and method to prevent unnecessary degradation. Other factors, including agitation speed, solvent concentration, and post-extraction treatments such as freeze–thaw cycles and storage conditions, can also indirectly influence polysaccharide structure [30,31,32,33]. Therefore, it is crucial to select appropriate extraction conditions to maximally preserve the native structural integrity of the HoPSs. Consequently, the structural alterations induced by these extraction variables necessitate precise and comprehensive analytical strategies to accurately elucidate the native or modified state of the HoPSs.

### 2.2. Analytical Techniques for HoPS Structure Assessment

Given the susceptibility of HoPSs to structural modification during extraction, as outlined above, the selection of appropriate analytical techniques is paramount. Advances in molecular biology and physicochemical analytical techniques have provided powerful tools for polysaccharide structural analysis [2]. Polysaccharide structures are typically categorized into primary and advanced (higher-order) structures. While current research often emphasizes primary structure elucidation, the advanced structure is more directly correlated with biological activity [1]. For HoPSs, primary structure analysis aims to determine the monosaccharide composition, glycosidic linkage types, chain length, and branching architecture. Monosaccharide identity and molar ratios are commonly analyzed by chromatography techniques such as gas chromatography (GC), high-performance liquid chromatography (HPLC), and ion chromatography (IC) [34]. Glycosidic linkage positions, branching patterns, sugar residue sequences, and anomeric configurations are characterized by methylation analysis, infrared spectroscopy (IR), mass spectrometry (MS), and nuclear magnetic resonance (NMR) spectroscopy [28,35]. Molecular weight and degree of polymerization are determined by size exclusion chromatography coupled with multi-angle laser light scattering (SEC-MALLS) and matrix-assisted laser desorption/ionization time-of-flight mass spectrometry (MALDI-TOF-MS) [36,37]. Analysis of advanced structures (secondary, tertiary, and quaternary) focuses on elucidating spatial conformation, aggregation state, and crystallinity. Circular dichroism (CD) spectroscopy is used to identify secondary conformations (e.g., β-sheets, triple helices) and monitor conformational dynamics in solution [38]. X-ray diffraction (XRD) determines crystalline forms and crystallinity [39]. Microscopy techniques—including atomic force microscopy (AFM), transmission electron microscopy (TEM), and scanning electron microscopy (SEM)—visualize nanoscale morphology (e.g., fibrous, spherical) and aggregation states [40]. Dynamic light scattering (DLS) and viscometry provide supplementary data on hydrodynamic size distribution, conformational rigidity, and aggregation stability in solution [41].

The methodological approach is often tailored to the biological origin of the HoPSs, as its structural features are intrinsically linked to its source and function. As summarized in Table 1, XRD is a core technique for characterizing the semi-crystalline structures of plant-derived HoPSs, where distinctions in glycosidic bond type (α-linked in starch vs. β-linked in cellulose) and branching (highly branched in amylopectin vs. linear in cellulose) are critical [42,43]. For animal-derived HoPSs such as chitin, analysis must quantify the degree of deacetylation and, when applicable, determine branched chain length using specific enzymatic assays. Advanced structure characterization focuses on the morphology of spherical aggregates (glycogen) or layered fibrous assemblies (chitin) [44,45]. Microbial HoPSs often exhibit amorphous organization. For instance, yeast-derived β-glucans possess a characteristic triple-helix conformation, whereas bacterial dextrans adopt a flexible random coil in solution; thus, CD spectroscopy is key for probing solution-phase conformation. Enzymatic digestion coupled with MS is essential for verifying branching patterns, and particular emphasis should be placed on correlating their network structures with bioactivity [46,47].

However, conventional one-dimensional techniques (e.g., 1D NMR, GC-MS) often struggle to resolve structural microheterogeneity, dynamic conformations, and complex branching topologies. This limitation underscores the need for emerging integrative approaches. Combining multi-dimensional NMR with computational modeling, employing small-angle X-ray scattering (SAXS) for solution structure, and utilizing advanced hyphenated techniques like HPAEC-PAD/MS are becoming essential. Such orthogonal methodologies are critical to deconvolute structural complexity, accurately determine parameters like helical persistence length and branching patterns, and thereby establish a robust foundation for mechanistic structure–activity relationship studies.

## 3. Structure-Immunomodulatory Activity Relationships of HoPSs

### 3.1. Monosaccharide Composition

Monosaccharide composition refers to the identity and molar ratio of the individual sugar units constituting a polysaccharide chain, serving as the fundamental chemical basis for its structural classification and biological recognition [10]. The monosaccharide composition of polysaccharides serves as a fundamental determinant of their immunomodulatory potential. Recent advances have elucidated the mechanisms of characteristic monosaccharides, the regulatory effects of monosaccharide ratios, and the targeting functions of specific sugars. These insights provide a crucial foundation for the rational design of HoPSs with enhanced immunobiological activities.

Glucose-based HoPSs remain a central focus. Curdlan, a linear β-(1→3)-D-glucan, induces the synthesis of cytokines such as IL-6, IL-1β, IL-23, IL-17, and IL-22 via the activation of Th1 and Th17 cells [51]. Curdlan and its derived gluco-oligosaccharides (GOSs) activate macrophages through CR3 (CD11b/CD18) and Toll-like receptor 2 (TLR2), engaging the MAPK and NF-κB pathways, positioning them as promising candidates for immunomodulatory functional foods [52]. A purified HoPS (CP2-3-1) from *Coptis chinensis*, predominantly composed of glucose, was shown to elevate serum levels of IgE, mMCP-1, and IL-4 antibodies in allergic mice. This immunoenhancement was linked to an increased richness and diversity of the intestinal microbiota [53]. Similarly, an exopolysaccharide (EPS) produced by *Bacillus tequilensis* PS21, containing only glucose, significantly stimulated neutrophil levels, serum lysozyme activity, and induced antioxidant enzymes (MPO, CAT, SOD) in Nile tilapia, highlighting its potential as an immunostimulant in aquaculture feed [54].

Other monosaccharides also confer distinct immunomodulatory properties. An EPS from *Parabacteroides distasonis*, composed solely of mannose, enhanced phagocytic activity and promoted the secretion of nitric oxide (NO) and pro-inflammatory cytokines (IL-1β, IL-6, TNF-α) in RAW 264.7 macrophages via NF-κB, MAPK, and Akt pathways [55]. Fucoidan SHC4-6, a sulfated mannan, exerted anti-inflammatory effects by inhibiting MAPK and NF-κB pathways, thereby improving skin barrier proteins and hydration factors [56]. Homogalacturonan (HG) from *Hippophae rhamnoides* berries activated macrophages via the TLR4/MyD88 pathway, increasing NO, IL-1β, and IL-6 production [57], while HG from okra demonstrated immunostimulatory activity on THP-1 macrophages [58]. Fungal-derived chitosan hydrogel inhibited pro-inflammatory factors (TNF-α, IL-1β, IL-6), promoted macrophage polarization towards the reparative M2 phenotype, and facilitated nerve repair and vascular regeneration [59].

Acidic monosaccharides, such as glucuronic acid, are generally associated with lower direct immune activity. However, specific levels of glucuronic acid can modulate immune regulation by altering polysaccharide solubility and charge characteristics, offering novel avenues for the study of acidic sugar-containing immunomodulators [60].

### 3.2. Molecular Weight

Molecular weight (MW) represents the hydrodynamic volume and chain length of a polysaccharide, which critically governs its solubility, rheological properties, and capacity to interact with cellular receptors [10]. Molecular weight is a critical structural determinant of the immunomodulatory activity of polysaccharides. The relationship between molecular weight and immune function is often non-linear, as distinct molecular weight ranges can modulate biological activity through differential effects on spatial conformation, receptor binding affinity, and signaling pathway activation. More and more research has clarified the optimal molecular weight ranges and their underlying regulatory mechanisms for various HoPSs.

For instance, high-molecular-weight oat β-glucan exhibits reduced immunostimulatory capacity, likely due to its high viscosity limiting bioaccessibility, whereas lower-molecular-weight fractions demonstrate superior immune activity and greater efficacy in suppressing cancer cell viability [61]. Similarly, the immunostimulatory effect of chitosan on RAW 264.7 macrophages is strongly molecular-weight-dependent. While both high- and low-molecular-weight chitosan enhance macrophage function via the NF-κB and AP-1 pathways, lower-molecular-weight chitosan shows greater potency [62]. In the case of λ-carrageenan, lower-molecular-weight fractions not only induce pro-inflammatory cytokines but also stimulate secretion of the anti-inflammatory cytokine IL-10, resulting in the highest overall cytokine induction efficiency—indicating that molecular weight reduction can confer unique immunomodulatory properties [63].

Further examples illustrate this nuanced relationship. A slight increase in the molecular weight of a galacturonic acid-rich polysaccharide from Schisandra chinensis fruit significantly enhances nitric oxide (NO) secretion and upregulates levels of TNF-α and IL-6, thereby boosting immune activation [64]. Conversely, medium-molecular-weight fucoidan (50–100 kDa) from Cladosiphon okamuranus enhanced natural killer (NK) cell activity by 28–32% and reduced the secretion of the pro-inflammatory mediator PGE_2_ by 40% (*p* < 0.05), thereby strengthening host immune defense [65]. These findings collectively underscore that the molecular weight–activity relationship varies substantially among different types of HoPSs, highlighting the importance of molecular weight optimization in the rational design of polysaccharide-based immunomodulators.

However, the relationship between molecular weight (MW) and immunomodulatory activity is not linear. While low-MW polysaccharides offer better solubility, high-MW β-glucans often exhibit superior immunostimulatory effects [58]. These contradictions likely stem from the MW’s impact on conformation. Low MW may disrupt the triple helix or reduce receptor clustering avidity, whereas excessively high MW limits bioaccessibility due to viscosity. Thus, the optimal MW is highly dependent on the specific polysaccharide type and its native conformation.

### 3.3. Glycosidic Bond Types and Linkage Patterns

Glycosidic bond types and linkage patterns describe the stereochemistry (α or β configuration) and connectivity positions (e.g., 1→3, 1→4, 1→6) between adjacent sugar residues, dictating the backbone architecture and conformational flexibility of the polymer [10]. The type, configuration, linkage sites, and spatial arrangement of glycosidic bonds in HoPSs are core structural determinants of their immunomodulatory activity. These features influence polysaccharide conformation, modulate immune cell activation, and direct cytokine secretion, thereby shaping the strength and direction of the immune response.

β-linked glycosidic bonds are widely associated with potent immunomodulation. A β-(1→3)-linked backbone is particularly recognized for strong immune activity, often enhanced by β-(1→6)-linked side chains. For instance, a β-(1→3)-glucan from Russula vinosa Lindblad, bearing two β-glucosyl side chains at every fifth O-6 position, exhibits significant immunostimulatory activity [66]. Similarly, the β-(1→3)-glucan backbone of Dictyophora polysaccharide DIP, with β-1,4- and β-1,6-branching, more effectively suppressed pro-inflammatory factors, reducing IL-1β and IL-6 levels by 45.8% and 52.3%, respectively (*p* < 0.01), and repaired the intestinal mucosal barrier [67]. Terminal mannose residues in β-1,4-linked D-mannan may engage mannose receptors, playing a key role in innate immunity and pathogen recognition [68]. Additionally, a Dendrobium polysaccharide (DDP-1) with a main chain of →4)-β-D-Manp-(1→ and →4)-2-O-acetyl-β-D-Manp-(1→ enhances immune function by promoting TNF-α and IL-6 production in THP-1 macrophages [69].

α-linked glycosidic bonds, traditionally considered less immunogenic, are now recognized for distinct structure–activity relationships. Modifications such as C-glycosidic linkage (replacing O-glycosidic bonds) can improve enzymatic stability and prolong immune activity [70]. An α-(1→6)-D-glucan from banana enhances immunity by promoting T-cell proliferation, macrophage phagocytosis, CD3^+^ T-cell levels, and CD4^+^/CD8^+^ ratios, and elevating IL-6, IgG, IgM, TNF-α, and hemolysin antibodies [71]. Similarly, an α-(1→4)-linked glucan from Astragalus (APS-A1), with a 1,4,6-α-D-Glcp branch every tenth residue, inhibits inflammatory cytokines (TNF-α, IL-6, MCP-1) in macrophages via NF-κB and MAPK pathways, demonstrating anti-inflammatory activity [72].

Glycosidic bond sites and conformational effects critically influence backbone conformation, flexibility, and stability. The (1→3) linkage favors rigid helical structures, and β-(1→3)-glucans can adopt stable triple-helical conformations. The β-(1→3)-linked backbone of Dictyophora polysaccharide DIP forms a rigid rod-like structure due to steric constraints. Introducing (1→6) linkages into the main chain disrupts helical continuity, promoting a flexible random coil [65]. In contrast, (1→4) linkages tend to form linear, flexible chains. The dihedral angles in β-(1→4)-linked cellulose promote extended linearity, with individual chains exhibiting linear flexibility in aqueous solution [73]. Moreover, 1,2-cis-glycosidic bonds, such as 1,2-cis-xylosidic linkages, exhibit greater conformational flexibility, with the cis configuration being key to maintaining the overall flexibility of the glycan chain [74].

### 3.4. Spatial Conformation

Spatial conformation refers to the three-dimensional arrangement of the polysaccharide chain, ranging from random coils to ordered structures such as helices or sheets, which determines the topological compatibility with specific protein binding sites [10]. In recent years, research on the structure–activity relationship between the spatial conformation and immune activity of HoPSs has made a breakthrough. Advances in molecular dynamics simulations, receptor docking studies, and validation in cellular and animal models have consistently highlighted that the integrity and flexibility of helical conformations are pivotal determinants governing immune receptor binding and downstream signaling pathway activation. Distinct spatial conformations confer markedly different immunomodulatory mechanisms.

Ordered helices, particularly the triple helix, confer high structural stability and are strongly correlated with potent immunostimulatory activity. The triple-helix conformation, formed by rigid tertiary folding, exhibits superior resistance to environmental perturbation and consistently demonstrates higher immune and biological activity compared to its single-stranded or random coil counterparts [75]. Structural integrity is crucial. For instance, a continuous β-(1→3)-glucan chain from yeast can precisely dock into the binding domain of the Dectin-1 receptor, effectively activating the Syk/NF-κB pathway [76]. The structural parameters of the helix itself are also tunable. An increase in β-(1→6)-linked side chains on a β-(1→3)-glucan backbone can alter the helix pitch and expand its hydrophobic cavity, directly influencing molecular stiffness and receptor interaction [77]. This is exemplified by β-(1→3,1→6)-glucan from *Pleurotus sajor-caju* and a β-1,3-branched β-1,2-mannan from *Hericium erinaceus*, both of which significantly upregulated macrophage NO production by 3.5-fold and TNF-α secretion to 285.4 ± 20.1 pg/mL (*p* < 0.01) via their native triple-helical structures [78,79].

In contrast, flexible conformations such as random coils offer distinct advantages for immune modulation. Their lower steric hindrance and greater degrees of conformational freedom facilitate easier access to and interaction with immune cell surfaces or receptor clefts [80]. This enhanced accessibility can lead to potent immune activation. For example, a galactan in a random coil conformation interacts with the hydrophobic binding site of the TLR4/MD2 complex, triggering the TLR4/NF-κB pathway and stimulating macrophage proliferation, phagocytosis, and cytokine secretion [81]. Furthermore, the local chain dynamics and torsional flexibility within oligomeric or branched β-glucan random coils can enhance affinity for receptors like Dectin-1 by allowing for optimal “induced-fit” binding [82,83]. The spatial display of bioactive epitopes can also be more prominent in certain flexible conformations, allowing key motifs to interact more effectively with immune components.

### 3.5. Branch Structure

Branch structure characterizes the presence, density, and distribution of side chains attached to the main polysaccharide backbone, modulating the molecule’s accessibility to enzymes and influencing its overall bioactivity [10]. The degree of branching (DB) of HoPSs directly modulates its immunomodulatory activity by altering molecular conformation, aqueous solubility, the number of available receptor-binding sites, and the mode of interaction with immune cells. An optimal branching degree maximizes the activation of immune signaling pathways, while distinct types of polysaccharides exhibit different patterns of immune activity depending on their specific branching architecture and distribution.

β-(1→3,1→6)-glucans extracted from edible mushrooms, with a branching ratio of 1:3 to 1:4, exhibit the strongest binding affinity for Dectin-1 (binding efficiency: 85.2 ± 3.1%), leading to a 4.2-fold increase in NF-κB transcriptional activity. Conversely, a glucan with a 1:2 branching ratio showed a 60% reduction in receptor binding efficiency due to steric hindrance, drastically diminishing immune activity [50]. In contrast, a glucan with a 1:2 branching ratio (excessive branching) suffers from pronounced steric hindrance, which disrupts effective receptor binding and diminishes immune activity. Conversely, a glucan with a 1:9 ratio (insufficient branching) has a limited ability to activate immune receptors due to inadequate exposure of binding sites [50]. Lentinan (from *Lentinus edodes*) maintains a stable triple-helical conformation and sustains immune cell activation during in vitro fermentation when its DB is approximately 40%. Deviation from this optimal range reduces conformational stability and significantly impairs immunostimulatory activity [84]. Yeast-derived β-glucan acts as an optimal immune modulator with a branching ratio of 1:3–1:5; excessive branching reduces binding efficiency to immune receptors because of steric effects [85]. A relatively lower DB facilitates more efficient binding to Dectin-1, thereby activating the Syk-CARD9-NF-κB pathway and increasing nitric oxide (NO) production in macrophages [46]. Barley β-glucan, which has a DB < 15% and a predominantly linear structure, exhibits significantly weaker Dectin-1 binding affinity compared with yeast β-glucan (DB 20–40%). Consequently, it only partially activates the Dectin-1-mediated immune pathway, resulting in markedly lower immune activity [86,87].

Nevertheless, the immunomodulatory role of branching is not universally consistent across all HoPSs. While moderate branching (e.g., 1:3 to 1:4 for β-glucans) is widely reported to maximize Dectin-1 binding and immune activation [50], studies on glycogen reveal an inverse relationship. A lower degree of branching (~8.1%) potentiates macrophage activation, whereas hyper-branched structures (20%) suppress it [49]. This suggests that the functional consequence of branching is context-dependent, varying significantly between structural-support polysaccharides (like glycogen) and recognition-based immunomodulators (like yeast glucans).

The Dectin-1 receptor and β-glucan form a core recognition pair for specific immune activation, and their binding efficiency is directly governed by the dynamic and static structural features of the glucan. We summarize the binding patterns and comparative effects of glucans from different sources with the Dectin-1 receptor in Table 2. For fungal β-glucans, a higher DB significantly enhances binding efficiency and immune activity by stabilizing the active conformation and providing a multivalent binding interface. In contrast, linear or low-branched β-glucans from cereals and bacteria lack these structural characteristics, leading to substantially weaker receptor binding and immune activation.

The introduction of β-1,6-N-acetyl-D-glucosamine branches onto a β-(1→4)-N-acetyl-D-glucosamine backbone via glycosylation yields a branched derivative that is highly recognized and activated by macrophages, unlike natural linear chitin, which has a DB near zero [48]. Glycogen with a DB of ~8.1% potently activates macrophages to produce NO and TNF-α and can modulate the immune response within the tumor microenvironment. However, increasing the DB to 20% drastically reduces this immunostimulatory effect [49]. Compared with yeast β-glucan, chitin and glycogen possess lower DB values, reflecting their primary biological roles in structural support and energy metabolism rather than immune activation. Consequently, they do not require a high DB to expose receptor-binding sites. A lower DB allows these polymers to optimize their core functions while avoiding issues such as structural instability and reduced metabolic efficiency, exemplifying a functional–structural adaptation.

### 3.6. Chemical Modification

Chemical modification involves the controlled introduction of functional groups (e.g., carboxymethyl, acetyl, sulfate) to native polysaccharides, aiming to tailor their physicochemical properties and enhance specific pharmacological or nutritional effects [10]. These modifications influence immune activity in a structure-dependent manner, primarily by changing the degree of substitution and the spatial conformation of the HoPS.

Acetylation is a widely used method for modifying polysaccharides. The introduction of acetyl groups can adjust the hydrophobicity and spatial conformation of an HoPS, thereby facilitating its binding to receptors on immune cell surfaces. For example, acetylation at the O-2 and O-3 positions of the β-1,4-Manp glycosidic bond in Dendrobium polysaccharides enhances macrophage immune functions, including NO release and phagocytosis [88]. Compared with chitooligosaccharides, N-acetylchitooligosaccharides (NACOS) not only promote the production of inflammatory cytokines (IL-1β, IL-6, and TNF-α) but also significantly inhibit NO production, thereby improving the suppression of LPS-induced inflammatory responses in RAW264.7 cells [89]. Moreover, N-acetylation of the *Streptococcus agalactiae* serotype Ia capsular polysaccharide markedly enhances its antigenicity, leading to more effective activation of the murine immune system [90].

Sulfation modifies the charge properties and spatial conformation of HoPSs, with immunomodulatory activity closely associated with the degree of sulfation and the substitution sites. Sulfated glucan from Antrodia cinnamomea inhibits the TGF-β/FAK/AKT signaling pathway by inducing lysosome-dependent degradation of the TGF-β receptor, thereby enhancing immunity and suppressing the survival and migration of cancer cells [91]. Sulfated fucoidan from sea cucumber significantly enhances RAW264.7 macrophage activity, promotes reactive oxygen species (ROS) production, strengthens phagocytic capacity, and induces the secretion of numerous cytokines [92]. While moderate sulfation can augment immune cell functions, excessive sulfation may disrupt the core polysaccharide structure and weaken receptor-binding ability [93].

Carboxymethylation introduces carboxymethyl groups into the polysaccharide chain, altering its spatial structure and water solubility, which ultimately modulates immune activity. For instance, carboxymethylated *Gastrodia elata* glucan GEP-1 exhibited low cytotoxicity, with RAW264.7 cell viability remaining above 95% at concentrations up to 200 µg/mL [85]. Furthermore, GEP-1 markedly promoted the secretion of TNF-α and IL-6, reaching 156.3 ± 12.4 pg/mL and 312.7 ± 28.5 pg/mL, respectively, compared with 45.2 ± 6.8 pg/mL and 98.4 ± 10.2 pg/mL in the control group (*p* < 0.01) [94]. Although the biological activity of polysaccharides is closely linked to their structural features, not all chemical modifications substantially enhance immune activity. This may be attributed to the introduction of substituents at non-optimal positions, which can impair the functional conformation or receptor interaction [95].

### 3.7. Hierarchical Integration of Structural Determinants

**Figure 2:**
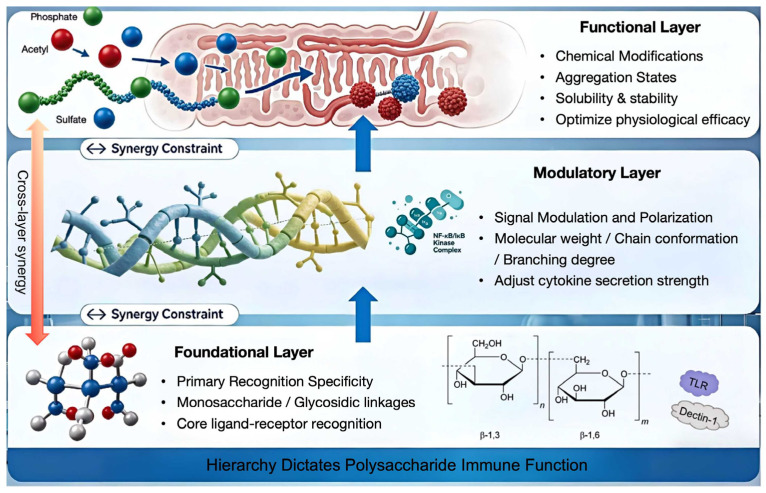
Hierarchical engineering framework of HoPS immunomodulatory activity.

While individual structural parameters are often discussed in isolation, their immunomodulatory effects are not merely additive but operate through a tightly coordinated hierarchy. Recognizing this layered architecture is critical to transcending empirical structure–activity relationships. The current literature supports a unified, three-tiered framework for understanding how polysaccharide structure dictates immune function [96] (Figure 2):

- Foundational Layer: Primary Recognition Specificity.

This layer comprises monosaccharide identity and glycosidic linkages, which dictate the fundamental ligand–receptor interaction. The specific interaction between polysaccharides and cellular receptors is highly dependent on structural features, where the configuration of the glycosidic bond determines whether the molecule is recognized as a ligand [97]. Molecular dynamics simulations confirm that the specific recognition between β-(1→3)-D-glucan and the Dectin-1 receptor, which is strictly governed by the monosaccharide type and the β-(1→3) linkage, forms a stable hydrogen-bond network essential for initiating downstream signaling [49,98]. In Figure 2, this layer is located at the bottom of the three-layer structure, which defines the primary recognition specificity of HoPSs, determined by monosaccharide composition and glycosidic linkages (e.g., β-1,3 and β-1,6 linkages in the depicted disaccharide repeat unit). Core ligand–receptor interactions are illustrated via TLR (Toll-like receptor) and Dectin-1 (C-type lectin receptor) engagement. This layer establishes the initial immune trigger. Blue arrows indicate upward propagation of regulatory effects.

- Modulatory Layer: Signal Modulation and Polarization.

Encompassing molecular weight (MW), conformation, and branching, this tier acts as a regulator for signal magnitude. Research indicates that physicochemical characteristics, such as MW and degree of branching, directly dictate immunomodulatory potency [99]. For instance, high-MW β-glucans activate macrophage surface receptors via rigid triple-helical conformations, which enhance receptor clustering avidity [50,99]. Furthermore, specific branching ratios (e.g., β-1→6/β-1→3) significantly fine-tune the activation of Dectin-1 and Toll-like receptors, balancing motif presentation with steric accessibility to modulate cytokine output without altering the core recognition logic [50]. In Figure 2, this layer is located in the middle of the three-layer structure, which mediates signal transduction and immune polarization via molecular parameters such as molecular weight, chain conformation, and branching degree. The triple-helix structure symbolizes conformational flexibility and dynamic structural transitions that influence receptor binding and downstream signaling. The NF-κB/IκB kinase complex is shown as a key signaling node regulating cytokine secretion strength. Blue arrows indicate upward propagation of regulatory effects.

- Functional Layer: Chemical Modifications and Aggregation States.

Chemical modifications and aggregation states refine the physiological efficacy of the polysaccharide. Evidence shows that targeted modifications such as sulfation effectively improve the solubility and bioactivity of natural polysaccharides [100], while specific structural refinements like the re-N-acetylation of capsular polysaccharides can significantly enhance vaccine immunogenicity [90]. They modulate solubility, enzymatic stability, and tissue tropism, bridging intrinsic activity with physiological efficacy. Additionally, the aggregation state influences tissue tropism and bioavailability, affecting how the molecule navigates the extracellular matrix [101]. In Figure 2, this layer is located at the top of the three-layer structure, representing the physiological outcomes of HoPS activity, including chemical modifications (e.g., acetylation, sulfation), aggregation states, solubility and stability, and ultimately optimized physiological efficacy. Green, red, and blue spheres denote phosphate, acetyl, and sulfate groups, respectively, which modulate surface properties and bioactivity.

Cross-layer synergy is paramount, as the efficiency of higher layers depends on the constraints established by the foundational structure. Methodologically, integrating this hierarchy into computational models is now feasible. Novel multi-scale structural information fusion methods provide a strategic guide for shifting the field from retrospective correlation to guide future glycoengineering [102]. In Figure 2, the vertical orange arrow labeled “Cross-layer synergy” indicates that functional outcomes emerge from integrated, non-linear interactions across all three layers. Bidirectional “Synergy Constraint” arrows between layers signify that each tier both influences and is constrained by adjacent layers—e.g., conformational changes in the Modulatory Layer can limit receptor accessibility in the Foundational Layer, while functional outcomes feed back to modulate structural stability. However, it must be acknowledged that the field currently lacks high-resolution structures of HoPS–receptor complexes (e.g., Dectin-1) and validated computational tools for in silico screening—gaps that must be filled to realize true predictive engineering.

The schematic illustrates a three-tiered structural hierarchy governing immune recognition and response:

- Foundational Layer (Bottom): Defines primary ligand–receptor specificity. It is determined by the monosaccharide composition and glycosidic linkages (e.g., β-1,3 and β-1,6 bonds in the disaccharide repeat unit). Interactions with immune receptors (TLR and Dectin-1) are shown as the initial trigger.
- Modulatory Layer (Middle): Regulates signal magnitude and polarization. Key parameters include molecular weight (Mw), chain conformation (e.g., triple-helix structure), and branching degree. The NF-κB/IκB kinase complex is depicted as the central signaling hub mediating cytokine secretion.
- Functional Layer (Top): Determines physiological efficacy. This layer integrates chemical modifications—green spheres (phosphate), red spheres (acetyl), and blue spheres (sulfate)—alongside aggregation states and solubility profiles.

Cross-layer synergy: The orange bidirectional arrow indicates that each layer both affects and is constrained by the adjacent layers. Blue arrows indicate upward propagation of regulatory effects.

## 4. Immunomodulatory Mechanisms of HoPSs

Chronic inflammation drives diverse global pathologies, yet current anti-inflammatory drugs face limitations in long-term safety and tolerability. This underscores the urgent demand for safer, naturally derived immunomodulators with sustainable application potential. Homopolysaccharides (HoPSs) have emerged as ideal candidates to fulfill this role, as their biological efficacy is intrinsically dictated by their structural architecture. The structure of an HoPS determines its immune recognition targets and downstream signaling pathways. Its immunomodulatory effects are primarily mediated through two interrelated mechanisms: direct interaction with immune cells, and indirect regulation of the gut microbiota and mucosal immunity. Together, these mechanisms modulate the balance between innate and adaptive immunity. In response to infection, polysaccharides can enhance immune surveillance and the clearance efficiency of pathogens and tumor cells by activating pro-inflammatory pathways. Conversely, in conditions of autoimmunity or allergy, polysaccharides can mitigate excessive inflammation and tissue damage by engaging anti-inflammatory pathways [103].

**Figure 3:**
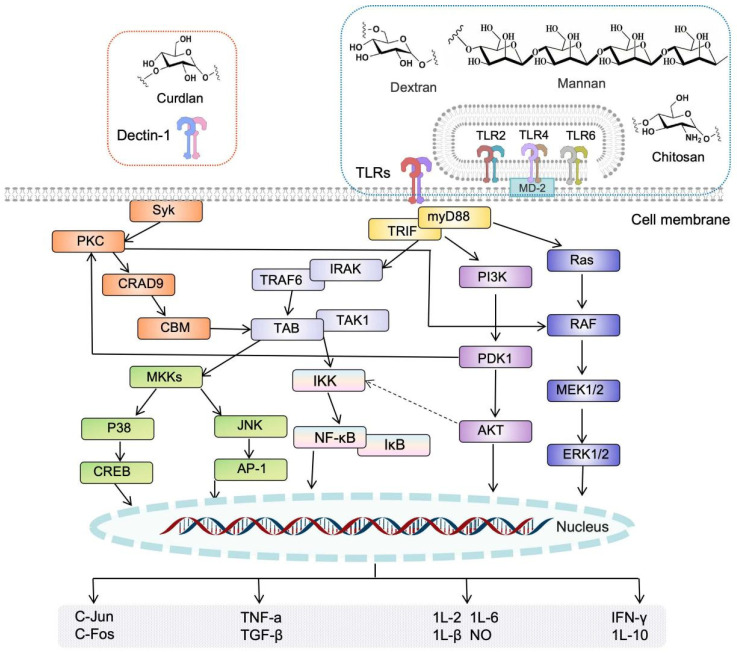
Schematic representation of HoPS-activated immune signaling pathways. (**Top**) Ligand–receptor engagement: curdlan binds Dectin-1, whereas dextran, mannan, and chitosan primarily engage the TLR2/4/6 complex. (**Middle**) Downstream signaling bifurcates via adaptor proteins: Dectin-1 utilizes Syk/CBM/PKC and TRAF6, while TLRs branch into MyD88-dependent (MAPK/ERK, PI3K/AKT) and TRIF-dependent pathways. Key kinases (IRAK, TAK1, IKK) and crosstalk mechanisms (e.g., AKT-mediated IKK inhibition) are shown. (**Bottom**) Activation of transcription factors (NF-κB, AP-1, CREB) induces nuclear translocation and subsequent production of inflammatory (TNF-α, IL-6, IL-1β, NO) and regulatory (IL-10, TGF-β) cytokines.

The immunomodulatory process of natural polysaccharides follows core principles involving receptor-mediated recognition, intracellular signal transduction, and the resulting immune response. As pathogen-associated molecular patterns (PAMPs), HoPSs are primarily recognized by pattern recognition receptors (PRRs), which constitutes the initial step in immune activation. Upon binding, the receptor transmits the signal intracellularly via cascades that ultimately regulate the expression of cytokines, chemokines, and cell surface markers. The core signaling pathways of HoPS immunoregulation can be broadly categorized into three major types, with the NF-κB pathway serving as a common convergence point. Following recognition by TLR2/4, polysaccharides activate the IKK (IκB kinase) complex via the MyD88/TRIF adaptor proteins, leading to phosphorylation and degradation of IκB-α. This promotes nuclear translocation of NF-κB (p65/p50), which subsequently drives the transcription of pro-inflammatory factors such as IL-1β, TNF-α, IL-6, and IL-12 [104,105]. Large-particulate or highly cross-linked polysaccharides tend to activate the NF-κB pathway, mediating pro-inflammatory and immunostimulatory effects. In contrast, low-molecular-weight or soluble polysaccharides often inhibit overactive NF-κB, helping to maintain immune homeostasis [12,106]. HoPSs also induce phosphorylation and activation of MAPK family members, including extracellular regulated kinase (ERK), c-Jun N-terminal kinase (JNK), and p38. Phosphorylated ERK, JNK, and p38 translocate to the nucleus, where they target and activate downstream transcription factors such as activator protein-1 (AP-1) and cyclic adenosine monophosphate response element-binding protein (CREB), initiating the transcription and expression of immune-related genes [107]. In the Dectin-1/Syk/CARD9 pathway, C-type lectin receptors (CLRs) such as Dectin-1 recognize ligands like β-glucan, and recruit and activate spleen tyrosine kinase (Syk). Activated Syk phosphorylates and activates phospholipase C (PLC), catalyzing the production of diacylglycerol (DAG) and inositol trisphosphate (IP3), and subsequently activating protein kinase C (PKC). CARD9 then mediates the activation of NF-κB and AP-1, triggering a potent pro-inflammatory response and promoting the polarization of Th17 cells [83,84]. The immunomodulatory activity of HoPSs is highly structure-dependent, and polysaccharide characteristics (e.g., molecular size, branching pattern, charge) determine which signaling pathway predominates. Figure 3 illustrates the integrated immune signaling network triggered by HoPS recognition. (Top Panel) Ligand–receptor interactions: curdlan activates Dectin-1, while dextran, mannan, and chitosan stimulate the TLR2/4/6 complex. (Middle Panel) The signaling networks are color-coded and bifurcated based on the adaptor proteins utilized: The Dectin-1 pathway (orange) proceeds via Syk and the CBM (CARD9–BCL10–MALT1) complex, activating PKC and TRAF6 (TNF receptor-associated factor 6). The TLR pathway splits into MyD88-dependent (purple/blue, leading to MAPK/ERK activation) and TRIF-dependent (yellow) branches. The diagram highlights key nodes such as IRAK (interleukin-1 receptor-associated kinase), TAK1 (TGF-β-activated kinase 1), IKK, and PI3K (phosphoinositide 3-kinase), demonstrating extensive crosstalk (e.g., AKT inhibiting IKK). (Bottom Panel) Nuclear translocation of transcription factors (NF-κB, AP-1, CREB) drives the expression of effector molecules including pro-inflammatory (TNF-α, IL-6, IL-1β, NO) and regulatory (IL-10, TGF-β) cytokines.

Despite the detailed signaling pathways described above, a major challenge in the field remains the methodological heterogeneity that obscures true structure–activity trends. The lack of standardized protocols for extraction, structural characterization, and bioassay—spanning different solvents, temperatures, and cell models (e.g., RAW264.7 vs. THP-1)—often yields incomparable results [66,89]. Moreover, resolving contradictions in polysaccharide immunomodulation requires moving beyond primary structures to integrate multi-scale structural analysis with in vivo outcomes, acknowledging that bioactivity loss is often mediated by conformational disruption rather than solely by chemical substitution.

The intracellular signaling cascades subsequently translate into cellular and humoral immune responses, mediating core functions such as anti-infection, anti-tumor, anti-inflammatory, and mucosal protection. Research shows that polysaccharides can robustly activate innate immunity. Macrophage phagocytic capacity was significantly enhanced by 2.5-fold (*p* < 0.01), accompanied by a 3.1-fold increase in NO production and a 40% elevation in lysozyme release, thereby markedly improving pathogen clearance efficiency [55]. Epithelial cells increase secretion of mucus, defense factors, and tight junction proteins (e.g., ZO-1 expression upregulated by 1.8-fold), leading to a more robust intestinal and respiratory mucosal barrier [108]. Dendritic cells (DCs) upregulate surface expression of MHC-II, CD80/CD86, and CD40 molecules, enhancing antigen presentation and subsequent adaptive immune activation [109]. Adaptive immunity is regulated in a polysaccharide-structure-specific manner. Large, particulate polysaccharides or β-glucans tend to promote the proliferation of Th1/Th17 cells, elevating serum IL-17A levels to 185.4 ± 15.2 pg/mL [110]. In contrast, small-particle polysaccharides, chitosan, or oligosaccharides favor the activation of Treg/Th2 cells, increasing the proportion of splenic Treg cells by 28% and elevating anti-inflammatory IL-10 secretion by 2.2-fold (*p* < 0.05), thereby exerting anti-allergic and tissue-repair effects [111]. Polysaccharides can also act as adjuvants, enhancing antigen-specific IgG and IgA antibody levels while reducing IgE levels to alleviate allergic responses. Notably, they strengthen IgA-mediated immunity in intestinal and respiratory mucosal areas, bolstering local defense [112].

Polysaccharides with low or negligible direct immunostimulatory activity (e.g., fructans, α-glucans, low-molecular-weight mannans) primarily exert their immunomodulatory functions via the microbiota–metabolite–immune axis. This also serves as an important auxiliary pathway for all polysaccharides, including highly active glucans, to enhance or fine-tune immune effects [11,13]. Upon fermentation by the gut microbiota, these polysaccharides not only modulate community structure but also drive intricate cross-feeding interactions between bacterial species (e.g., primary degraders and secondary consumers like Bifidobacterium and Lactobacillus), stabilizing the ecosystem and improving dysbiosis [113]. The functional outputs of this fermentation, termed postbiotics (including SCFAs, vitamins, and cell-free supernatants), exert direct immunomodulatory effects independent of the living microbe [114]. Specifically, SCFAs such as acetate, propionate, and butyrate activate specific G protein-coupled receptors (GPR41, GPR43, and GPR109A) on intestinal epithelial and immune cells; for instance, GPR43 signaling promotes Treg proliferation and IL-10 secretion, while GPR109A activation enhances barrier integrity [115,116]. Consequently, SCFAs strengthen the intestinal barrier by upregulating tight junction proteins (e.g., ZO-1, occludin) and maintain immune homeostasis. However, a critical translational gap remains, as in vitro fermentation models often fail to recapitulate the complex hydrodynamics, mucus interactions, and host-specific receptor profiles present in vivo, necessitating cautious extrapolation of in vitro metabolic profiles to human outcomes [117].

## 5. Prospects

While the structure–activity relationships of polysaccharides, such as the connections between molecular weight, glycosidic bonds, advanced conformation, and immunomodulatory activity, have been preliminarily established, challenges remain, including limitations in structural characterization and incomplete elucidation of their mechanisms of action. Future research is expected to advance in the following key directions: precise structural analysis, in-depth mechanistic studies, targeted modification technologies, and the expansion of diversified application scenarios.

### 5.1. Developing Advanced Technologies to Enhance Structural Elucidation

Characterizing the fine structure of HoPSs is fundamental to understanding and optimizing their biological activity. Emerging analytical technologies will push the boundary of what is resolvable. The development of ultra-high-field NMR (e.g., ≥1 GHz) coupled with innovative mathematical deconvolution algorithms and pulse sequences like pure shift and non-uniform sampling (NUS) will allow for the complete assignment of complex spectra from polysaccharides [118]. Advanced techniques such as Dynamic Nuclear Polarization (DNP)-NMR, which enhances sensitivity by orders of magnitude, will enable the detailed study of polysaccharide–protein interaction interfaces, particularly at the binding sites of receptors like Dectin-1 or TLR2 [119]. Surface-Enhanced Raman Scattering (SERS) combined with isotope labeling could provide unprecedented detail on glycosidic bond conformations and dynamics [120]. Single-molecule techniques, including high-speed atomic force microscopy (HS-AFM), can visualize the real-time conformational transitions of β-glucans (e.g., triple helix to random coil) under physiological conditions [121]. Integrating these experimental data with multi-scale molecular dynamics simulations and machine learning models will allow for the predictive modeling of structure–function relationships, such as how a specific branch point influences the stability of the active conformation. Glycomics approaches, such as liquid chromatography–tandem mass spectrometry (LC-MS/MS) with advanced fragmentation methods, will enable the sequencing of complex polysaccharide chains, including the sequencing of repeating units and identification of rare modifications [122].

### 5.2. Exploring Deep Mechanisms and Expanding Understanding of Immune Regulatory Networks

Future research must transcend the study of isolated signaling pathways to elucidate the integrated immune-regulatory networks orchestrated by HoPSs. A primary focus will be the “microbiota–metabolite–immune axis.” This involves using gnotobiotic animal models and in vitro human gut microbiome simulators to mechanistically dissect how HoPSs of defined structure (e.g., specific glycosidic linkages, branching patterns) selectively enrich for distinct bacterial consortia [123]. Subsequent metabolomics will link these shifts to the production of not only SCFAs but also other immunologically active metabolites like tryptophan derivatives, bile acids, and polyamines [124]. Single-cell RNA sequencing (scRNA-seq) of immune cells from the gut lamina propria, mesenteric lymph nodes, and systemic sites will reveal how these microbiome-derived signals, in concert with direct receptor engagement, program the differentiation and function of macrophages, dendritic cell subsets, and T cell populations (e.g., Treg, Th17, Th1) [125]. Advanced techniques like phosphoproteomics and spatial transcriptomics will be crucial for mapping these dynamic, cell-type-specific signaling events, building a comprehensive, predictive model of HoPS immunomodulation [126]. These systems biology approaches will explain inter-individual variability in response to polysaccharide interventions.

### 5.3. Optimizing Modification Techniques for Precise Immunoregulation

The future of HoPS modification lies in moving from semi-empirical approaches to rational, precision engineering. For targeted immunomodulation, modification strategies can be precisely tailored. For cancer patients, site-selective sulfation or phosphorylation may be explored to endow HoPSs with STING-activating capacity, as certain natural polysaccharides such as chitosan have been shown to induce type I IFNs via the cGAS–STING pathway [127]. Alternatively, polysaccharides can be engineered as carriers for checkpoint inhibitors (e.g., anti-PD-1 nanobodies) to achieve targeted delivery to the tumor microenvironment [128]. The future will also see increased adoption of green modification technologies, and enzymatic synthesis and modification will become dominant. Engineered glycosyltransferases, sulfotransferases, and acetyltransferases can catalyze highly regio- and stereo-specific modifications under mild conditions, eliminating the need for toxic reagents and protecting environmentally sensitive functional groups [129]. Microbial cell factories, such as engineered E. coli or Bacillus subtilis, can be programmed to produce structurally defined, pre-modified polysaccharides directly through synthetic biology approaches [130]. However, the transition from laboratory-scale biosynthesis to industrial-scale production presents significant hurdles. Scaling up microbial fermentation processes is often hampered by increasing viscosity leading to oxygen transfer limitations, genetic instability of engineered strains under prolonged culture, and the high cost of nutrient media, which collectively impede cost-effective commercialization [131].

### 5.4. Promoting Industrialization and Expanding Application Scenarios

Translating HoPS research into clinical and commercial applications requires a focus on standardization, formulation, and combination strategies. Integrating polysaccharides with vaccines, chemotherapeutic agents, and probiotics represents a promising direction, where modified polysaccharides can serve as vaccine adjuvants or synergize with drugs to reduce toxicity and improve therapeutic outcomes [132,133,134]. Beyond direct bioactivities, the structural versatility of HoPSs makes them ideal renewable building blocks for manufacturing complex nanomaterials and advanced biomaterials. Recent advances include gum polysaccharide-based nanocomposites fabricated through green approaches for drug delivery, food packaging, and tissue engineering [40], as well as pectin-based hydrogels that exhibit tunable mechanics, biocompatibility, and stimuli-responsiveness for smart drug delivery, wound dressing, and functional materials [135]. Developing symbiotic formulations that pair a specific prebiotic polysaccharide with a probiotic strain whose growth it preferentially stimulates, and which in turn produces a desired immunomodulatory metabolite (postbiotic), represents a powerful synergistic strategy for gut–immune axis modulation [136]. However, a critical translational gap remains. Current research severely lacks dose–response relationships and long-term human safety data; most in vivo studies rely on single, high rodent doses, failing to mimic chronic, low-dose human exposure. Future efforts must integrate pharmacokinetics, toxicology, and clinical trial design to establish standardized protocols for evaluating bioavailability and systemic distribution. Addressing these gaps is essential for translating precision engineering into clinical applications.

## 6. Conclusions

There exists a well-defined structure–activity relationship between the immunomodulatory activity of natural HoPSs and their structural characteristics, which is complex, specific, and synergistic. This review distills a core conclusion: the immunomodulatory potential of natural HoPSs is not dictated by isolated structural features, but arises from a hierarchical organization spanning primary recognition, conformational tuning, and functional bioavailability. While molecular weight, glycosidic linkages, and branching remain important, their effects are context-dependent and interactive, underscoring the limitations of simplistic structure–activity correlations. By integrating primary recognition, conformational modulation, and chemical refinement into a unified hierarchical model, this review summarizes a hierarchical framework aimed at transitioning HoPS research from empirical correlation to mechanism-informed glycoengineering, laying the groundwork for the rational design of immunotherapies and functional nutrition. Future advancements in high-resolution structural elucidation, multi-omics mechanistic dissection, and precision modification technologies are imperative to bridge the current translational gap. Ultimately, aligning structural complexity with nutritional and metabolic health performance will define the next phase of HoPS research, enabling their safe and effective application in functional foods, nutraceuticals, and clinical nutrition. This will provide robust theoretical support and technical assurance for the effective application of natural HoPSs in immune health. 

## Figures and Tables

**Table 1:** Structural characterization of representative HoPSs from different sources.

Name	Classifications	Structural Characteristics	Primary Structural Characterization	Advanced Structural Characterization	References
Starch	Plant (cereals, tubers, legumes)	Amylose is a linear α-1,4-glucan that adopts a left-handed helix, while amylopectin contains α-1,4/α-1,6 branches and forms dense, globular clusters.	Monosaccharide Composition: HPLC (post-hydrolysis). Glycosidic Linkage & Branching: Methylation GC-MS + NMR. Molecular Weight & Branching: SEC-MALLS + Iodine Binding Method.	Crystalline Structure & Crystallinity: XRD Conformation & Thermal Properties: CD + Differential Scanning Calorimetry (DSC) Morphology & Topography: SEM + AFM + SAXS	[9,42]
Cellulose	Plant (cotton, wood, other plant fibers)	Cellulose consists of linear β-1,4-glucose chains that are rotated 180° between residues. Intermolecular hydrogen bonding stacks these chains into rigid, parallel microfibrillar bundles.	Monosaccharide Composition: Acid Hydrolysis + GC-MS/HPLC. Glycosidic Linkage: Methylation GC-MS + FTIR + NMR. Molecular Weight & Conformation: SEC-MALLS.	Crystalline Structure & Crystallinity: XRD Conformation & Thermal Properties: CD + Raman spectroscopy Orientation & Fibril Structure: Polarized light microscopy	[41,43]
Chitin	Animal (crustacean shells, insect exoskeleton)	Chitin is a linear β-1,4-linked N-acetyl-D-glucosamine polymer. Its chains form stacked layers via hydrogen bonding, typically adopting an α-crystalline polymorph. Chitosan is its deacetylated derivative.	Monosaccharide Composition: Strong Acid Hydrolysis + HPLC. Glycosidic Linkage: Methylation GC-MS + FTIR. Degree of Deacetylation: ^1^H-NMR + Potentiometric Titration.	Crystalline Structure & Polymorph Identification: XRD + Thermogravimetric Analysis (TGA) Morphology & Topography: SEM + AFM + SAXS Hydrogen Bonding Network & Molecular Conformation: 13C-NMR	[44,48]
Glycogen	Animal (liver, muscle)	Glycogen consists of α-1,4-glucose chains with dense α-1,6 branching, forming highly branched, amorphous spherical aggregates.	Monosaccharide Composition: Acid Hydrolysis + GC-MS Glycosidic Linkage & Branching: FTIR + NMR Molecular Weight & Conformation: SEC-MALLS	Morphology & Topography: AFM + TEM + SAXS Solution Hydrodynamics: DLS + DSC + Gel Permeation Chromatography -Multi-Angle Laser Light Scattering (GPC-MALLS)	[45,49]
β-glucan	Microbial (Fungus)	Curdlan is a β-1,3-glucan (a polymer of glucose) with β-1,6 branches, adopting a triple-helix or mesh structure with low crystallinity (<10%).	Monosaccharide Composition: Acid Hydrolysis + GC-MS Glycosidic Linkage: Methylation Analysis + NMR Molecular Weight & Conformation: SEC-MALLS + MALDI-TOF-MS	Solution Conformation: CD + Raman spectroscopy + DSC Crystal Form & Crystallinity: XRD Morphology & Topography: SEM + AFM + TEM	[46,50]
Dextran	Microbial (Bacterium)	Dextran is an α-1,6-glucan (a polymer of glucose) with minor branching (α-1,3/1,4/1,2), adopting irregular, flexible coils rather than triple-helical structures.	Monosaccharide Composition: Acid Hydrolysis + HPLC/GC Glycosidic Linkage: Methylation Analysis + NMR Molecular Weight & Conformation: SEC-MALLS	Spiral Conformation: CD + MALLS-GPC + Optical Rotation (OR) Crystal Form & Crystallinity: XRD Morphology & Topography: TEM + AFM	[47]

**Table 2:** Comparative mechanisms of immunomodulation by β-glucans via Dectin-1.

Source	Interaction Mode with Dectin-1	Binding Affinity	Core Pathway	Main Mechanistic Limitations	Reasons for Key Differences
Yeast β-glucan	Specific binding to the extracellular C-type lectin-like domain (CTLD) of Dectin-1 via hydrophobic interactions, facilitated by the triple-helical conformation.	High (Reference: 100%)	Activation of Syk-CARD9-NF-κB signaling promotes inflammatory cytokine production, phagocytosis, and NK cell activity.	1. Poor bioavailability: High molecular weight (>1000 kDa) limits systemic absorption and bioavailability. 2. Context-dependent efficacy: Maximal immunomodulation often requires co-factors.	(1→3)-β-D-glucan backbone with (1→6)-β-linked branches (branching degree: 20–30%). The stable triple-helix structure binds well with Dectin-1.
Mushroom β-glucan	Binds to the carbohydrate-recognition site of Dectin-1 primarily via the linear backbone, enhancing binding affinity and stability.	Moderate to high (80–90%)	Dectin-1/TLR2/4 pathway activates DC maturation, antigen presentation, and Th1 response.	1. Source variability: Significant batch-to-batch variation in biological activity. 2. Thermal lability: High-temperature processing disrupts the triple-helical conformation.	Linear (1→3)-β-glucan backbone with variable (1→6)-β branches. Branching degree and pattern are species-dependent.
Cereal β-glucan	Weak, non-specific interaction with Dectin-1, primarily via hydrogen bonding or van der Waals forces.	Low (20–30%)	Inducing immune regulatory responses by regulating gut microbiota composition and producing SCFAs.	1. Weak direct immunostimulation: Minimal activation of innate immune cells. 2. The therapeutic effect of immune compromised state is limited.	Linear, mixed-linkage (1→3,1→4)-β-D-glucan. Low branching (<5%) and absence of stable triple helix.
Bacterial β-glucan	Binds Dectin-1 indirectly and weakly via aggregated or gel-formed triple-helical conformers. Linear chain lacks high-affinity multivalent binding sites.	Low to moderate (20–50%)	As a TLR2/TLR4-dependent adjuvant, it weakly activates the Dectin-1-Syk-CARD9-NF-κB axis and induces moderate cytokine/ROS.	1. Low solubility in physiological conditions limits bioavailability and cellular exposure. 2. Only triple-helical aggregates show detectable immune stimulation.	Linear, unbranched (1→3)-β-D-glucan. Bioactive triple-helix forms only upon heating/cooling or aggregation.

## Data Availability

The data presented in this study are available on request from the corresponding author.
